# Ultrahypofractionation in postoperative radiotherapy for breast cancer: A single‐institution retrospective cohort series

**DOI:** 10.1002/cam4.7367

**Published:** 2024-07-05

**Authors:** Angel Calvo Tudela, María Jesús García Anaya, Salvador Segado Guillot, Nuria Martin Romero, María Jesús Lorca Ocón, José Antonio Medina Carmona, Jaime Gómez‐Millán, Isabel García Ríos

**Affiliations:** ^1^ Department of Radiation Oncology Virgen de la Victoria University Hospital Malaga Spain; ^2^ Malaga Biomedical Research Institute Malaga Spain

**Keywords:** breast cancer, clinical observations, radiation therapy, radiotherapy

## Abstract

**Background:**

The ‘FAST‐forward’, study published in April 2020, demonstrated the effectiveness of an extremely hypofractionated radiotherapy schedule, delivering the total radiation dose in five sessions over the course of 1 week. We share our department's experience regarding patients treated with this regimen in real‐world clinical settings, detailing outcomes related to short‐term toxicity and efficacy.

**Methods:**

A descriptive observational study was conducted on 160 patients diagnosed with breast cancer. Between July 2020 and December 2021, patients underwent conservative surgery followed by a regimen of 26 Gy administered in five daily fractions.

**Results:**

The median age was 64 years (range: 43–83), with 82 patients (51.3%) treated for left‐sided breast cancer, 77 patients (48.1%) for right‐sided breast cancer, and 1 instance (0.6%) of bilateral breast cancer. Of these, 66 patients had pT1c (41.3%), 70.6% were infiltrative ductal carcinomas, and 11.3% were ductal carcinoma in situ. Most tumours exhibited intermediate grade (41.9%), were hormone receptor positive (81.3%), had low Ki‐67 (Ki‐67 < 20%; 51.9%) and were Her 2 negative (85%). The majority of surgical margins were negative (99.4%). Among the patients, 72.5% received hormonotherapy, and 23.8% received chemotherapy. Additionally, 26 patients (16.3%) received an additional tumour boost following whole breast irradiation (WHBI) of 10 Gy administered in five sessions of 2 Gy over a week. The median planning target volume (PTV) was 899 cm^3^ (range: 110–2509 cm^3^). Early toxicity was primarily grade I radiodermatitis, affecting 117 patients (73.1%). During a median follow‐up of 15 months (range: 3.9–28.77), only one patient experienced a local relapse, which required mastectomy.

**Conclusions:**

The implementation of this highly hypofractionated regimen in early‐stage breast cancer appears feasible and demonstrates minimal early toxicity. However, a more extended follow‐up duration would be required to evaluate long‐term toxicity and efficacy accurately.

## Graphical abstract

An ultra‐hypofractionated radiotherapy schedule is safe and effective after breast‐conserving surgery. 26 Gy in five fractions shows low acute toxicity. Most patients had grade one radiodermitis (73.1%). This scheme has important clinical benefits for patients who are frail o live far from the hospital in addition to economic advantages.

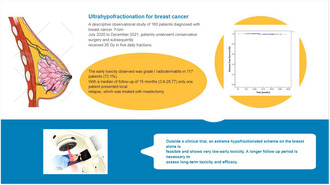

## 1 INTRODUCTION

Various studies have shown that for patients with early breast cancer, treatment with radiotherapy after breast‐conserving surgery reduces the risk of any recurrence of breast cancer by a half, and of breast cancer‐related mortality by a sixth. A meta‐analysis showed that for each local recurrence prevented, one breast cancer‐specific mortality is avoided. The authors conclude that the standard treatment for local invasive breast cancer is breast‐conserving surgery followed by adjuvant radiation therapy.^1^

Traditionally, the standard treatment is to deliver a dose of 50 Gy in 2 Gy fractions over 5 weeks. However, recent decades have seen a growing interest in shortening the duration of conventional treatment with hypofractionated radiation therapy (HF‐RT), delivering higher daily doses to the entire breast, reducing the duration of treatment and increasing patient comfort. Different studies have shown that hypofractionation in breast cancer has a solid radiobiological and clinical background. Various randomised studies comparing HF‐RT with conventional fractionated treatment have reported equivalent results: A shorter treatment time, with lower cost and less toxicity, means that HF‐RT has become the standard adjuvant radiotherapy treatment in breast cancer.^2^, ^3^, ^4^, ^5^, ^6^, ^7^, ^8^, ^9^

The standard treatment for breast cancer after conservative surgery and axillary study is irradiation of the breast, which may be associated with systemic and/or hormonal therapy. Several randomised trials with long‐term follow‐ups have shown a reduction by half in the local recurrence rate after application of radiotherapy subsequent to conservative surgery.^10^, ^11^ Recent trials confirmed these results, showing that patients treated with moderate hypofractionation in 15 fractions presented similar results in terms of efficacy and toxicity.^3^, ^7^, ^12^, ^13^

In recent years, more extreme hypofractionation regimens have been investigated. The 10‐year results of “UK FAST” trial study were published in 2020. This study compared a classic scheme of 50 Gy in 25 fractions vs two different weekly hypofractionated regimens. This trial didn't show any significant differences in terms of normal tissue effects between the 28.5 Gy in 5.7 Gy fractions for 5 weeks and 50 Gy regimen.^14^ The “FAST‐forward” phase III trial was published in April 2020, comparing in patients with pT1‐3pN0‐1 M0 stage treated with surgery a scheme of 40 Gy in 15 fractions versus two different hypofractionated regimens. This study demonstrated that delivering a dose of 26 Gy in fractions of 5.2 Gy over the course of 1 week was non‐inferior to a 15‐fraction regimen.^15^ Following this publication and coinciding with the COVID pandemic, our department adopted the 26 Gy treatment schedule given the great interest in reducing treatment regimens in radiotherapy. The objective of this retrospective study was to evaluate the effectiveness and short‐term toxicity of this regimen within a standard clinical practice setting.

## 2 MATERIALS AND METHODS

We conducted a retrospective observational study on patients treated at our center using the FAST‐forward regimen, which involved delivering a total dose of 26 Gy in five fractions over 1 week. The study was approved by the local ethics committee (PEIBA), with the code GICOR‐2010‐01.

We assessed 160 consecutive patients treated between July 2020 and December 2021. Inclusion criteria were women over 40 years of age with invasive or in situ breast carcinoma after complete microscopic removal of the primary tumour by breast‐conserving surgery, classified according to the AJCC Cancer Staging Manual Eighth Edition.^16^ Exclusion criteria were mastectomized patients, cases of breast reconstruction, treatment with concomitant chemotherapy or indication for radiotherapy in nodal areas. All patients underwent breast‐conserving surgery, with either sentinel lymph node biopsy (SLNB) or axillary lymph node dissection (ALND), along with concurrent administration of endocrine therapy or trastuzumab. In the first consultation, the procedure was explained, and patients signed informed consent. All patients had undergone treatment planning that began with a supine computed tomography, performed using a standard breast tilt table with the patient immobilised.

The treatment protocol involved administering 26 Gy in five fractions of 5.2 Gy using the irradiation techniques outlined previously. Additionally, a sequential tumour bed radiotherapy boost (10 Gy in five fractions of 2 Gy) was delivered over the lumpectomy cavity, with patient selection left to the discretion of the treating physician.^17^ Target volume delineation followed ESTRO consensus guidance,^18^ with the clinical target volume (CTV) encompassing all breast gland tissue, excluding the ribs and pectoralis muscle. The planning target volume (PTV) includes the CTV with a margin of 5 mm. Anatomo‐clinical target volume coverage was between 95 and 100% of the prescribed dose. In accordance with the FAST‐Forward protocol, the dose constraints for organs at risk were: volume of ipsilateral lung receiving 8 Gy, less than 15%; volume of heart receiving 1.5 Gy, less than 30%, and volume receiving 7 Gy, less than 5%. Before each fraction, verifications were performed using electronic portal imaging. All patients received photon energies of 6 MeV with customised, forward planned, segment‐weighted approaches.

Patient follow‐up included clinical examination and assessment of acute toxicity throughout treatment, upon treatment completion, at 4 weeks post‐treatment, and subsequently every 6 months for a duration of 1 year, utilising the Common Terminology Criteria for Adverse Events (CTCAE version 4.0).^19^ The toxicity variables evaluated were: radiodermatitis, asthenia, fibrosis, breast pain, breast induration in the tumour bed, skin hyperpigmentation, breast oedema.

The end points for the statistical analysis were overall survival (OS) and progression free survival (PFS). OS was defined as the period between the diagnosis and the date of the death, and PFS was defined as the period between the diagnosis and locoregional progression, distant metastasis, or mortality from any cause. Actuarial survival was calculated using the Kaplan–Meier method. Statistical analyses were conducted using the Statistical Package for the Social Sciences (SPSS) version 20 for Windows (IBM, Chicago, IL, USA).

## 3 RESULTS

Table 1 presents the baseline characteristics of all 160 patients. The median age was 64 years (range 43–83 years). Eighty‐two patients (51.3%) received treatment for left breast and 77 patients (48.1%) for right breast cancer. In one patient (0.6%), the diagnosis was bilateral synchronous breast cancer.

**TABLE 1:** Patient characteristics.

Patient characteristics	*N* (%)
*Median age*	64 years (43–83)
*Age range*	
40–49	10 (6.3)
50–59	44 (27.5)
60–69	68 (42.5)
70–79	33 (20.6)
80–89	5 (3.1)
*Tumour grade*	
1	35 (21.9)
2	67 (41.9)
3	58 (36.2)
*Primary tumour side*	
Right	77 (48.1)
Left	82 (51.3)
Bilateral	1 (0.6)
*Pathological T stage*	
pTis	18 (11.3)
pT1a	13 (8.1)
pT1b	27 (16.9)
pT1c	66 (41.3)
pT2	36 (22.5)
*Pathological N stage*	
pNx	6 (3.8)
pN0	137 (85.6)
pN1mic	12 (7.5)
pN1	5 (3.1)
*Histological type*	
Ductal carcinoma in situ	18 (11.3)
Infiltrating ductal	113 (70.6)
Infiltrating lobular	21 (13.1)
Other	8 (5.1)
*Biological classification*	
Luminal A	83 (51.9)
Luminal B	45 (28.1)
Triple negative	8 (5)
Her2+ HR‐	1 (0.6)
Her2+ HR+	2 (1.3)
Unknown	21 (13.1)
*Lymphovascular invasion*	
Present	6 (3.8)
Absent	151 (94.4)
Unknown	3 (1.9)

In pathological T stage, most patients had pT1c (41.3%) and pT2 (22.5%). One hundred and thirty‐seven patients had pN0 (85.6%) in pathological lymph node stage. Regarding histological type the highest proportion were ductal infiltrative tumours (70.6%), lobular infiltrative (13.1%) and ductal carcinoma in situ (11.3%). Tumours were mainly intermediate grade (41.9%) with a 36.2% of high grade and 21.9% low grade. In biological classification most patients were luminal A (51.9%) and luminal B (28.1%). One hundred and fifty‐one patients (94.4%) had absence of lymphovascular invasion.

Table 2 shows details of patient treatment. Breast‐conservation surgery with SLNB was performed on 95.6% patients. Surgical resection margins were mainly negative (85%). The majority of patients received adjuvant hormonal therapy (72.5%). Thirty‐eight patients (23.8%) received chemotherapy, the majority with adjuvant intention (19.4%) and only three patients received anti‐HER2 treatment (1.9%).

**TABLE 2:** Treatment‐related variables.

Treatment‐related variables	*N* (%)
*Breast surgery*	
Breast‐conserving surgery with sentinel lymph node biopsy (SLNB)	153 (95.6)
Breast‐conserving surgery with axillary dissection (ALND)	2 (1.3)
Breast‐conserving surgery	5 (3.1)
*Surgical margin*	
Negative >2 mm	136 (85)
Close <2 mm	23 (14.4)
Positive	1 (0.6)
*Chemotherapy*	
Yes	38 (23.8)
No	122 (76.3)
*Intention of chemotherapy*	
Neoadjuvant	7 (4.4)
Adjuvant	31 (19.4)
*Anti‐HER 2 treatment*	
Yes	3 (1.9)
No	157 (98.1)
*Hormonal therapy*	
Yes	116 (72.5)
No	44 (27.5)
*Boost*	
Yes	26 (16.3)
No	134 (83.8)
*Genomic test*	
Yes	57 (35.6)
No	103 (64.4)

A sequential boost of 10 Gy in five sessions of 2 Gy after whole breast irradiation (WHBI) was administered to 26 patients (16.3%).

The median volume of the planning target volume (PTV) was 899 cm^3^, ranging from 110 to 2509 cm^3^.

**FIGURE 1:**
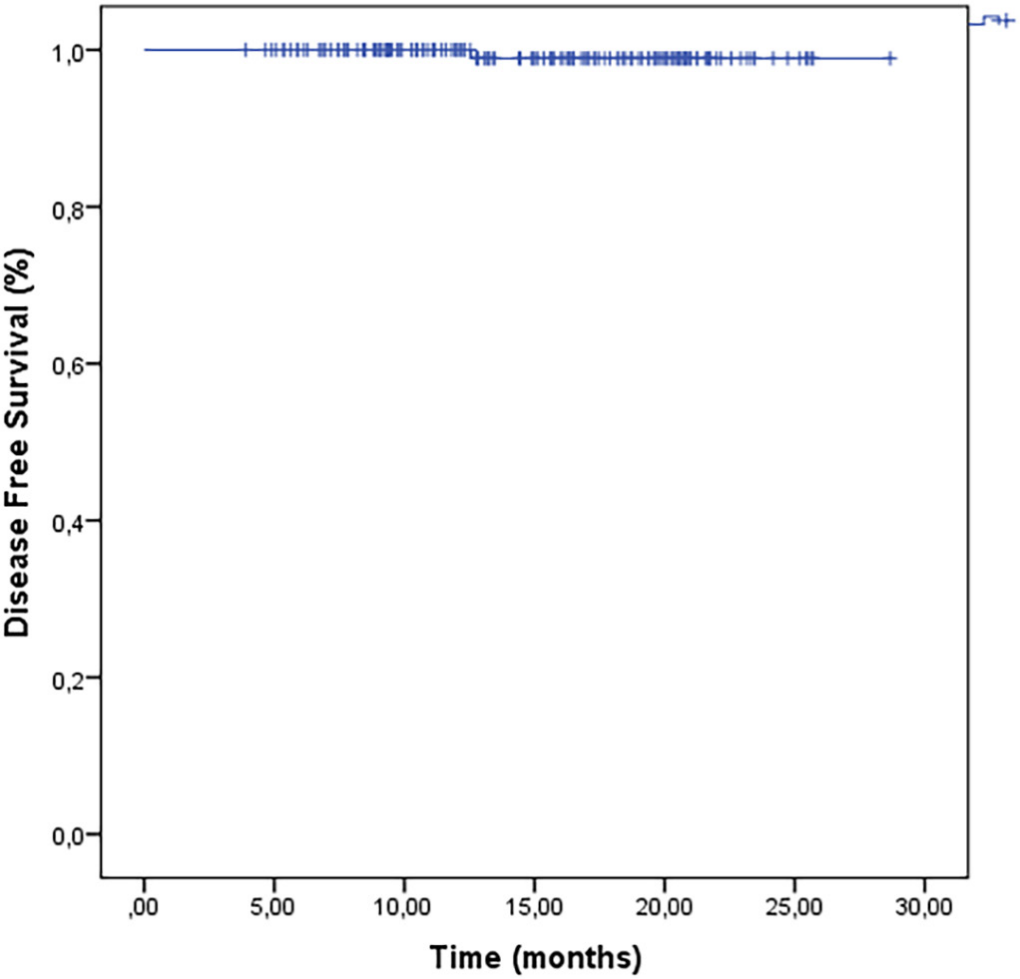
Disease‐free survival.

With a median follow‐up duration of 15 months (range: 3.9–28.77 months), exclusive local recurrence occurred in one patient (0.6%), who underwent salvage radical mastectomy. At the time of writing, 160 patients (100%) were alive and disease‐free, with 2‐year OS and disease‐free survival (DFS) rates of 100% and 98.1% respectively (Figure 1).

Tolerance to radiotherapy was excellent (Table 3). Radio‐dermatitis was G0 in 34 patients (21.3%), G1 in 117 (73.1%), G2 in 8 (5%) and G3 in one (0.6%). The rates of grade 1 or 2 radio‐induced asthenia were 21.3% and 1.9% respectively, while the majority of patients were grade 0 (76.9%). The cases of acute toxicity were resolved a month after finishing the treatment. No patient was COVID‐positive during radiotherapy treatment.

**TABLE 3:** Toxicity.

Toxicity	*N* (%)
*Radio‐dermatitis*	
G0	34 (21.3)
G1	117 (73.1)
G2	8 (5)
G3	1 (0.6)
*Asthenia*	
G0	123 (76.9)
G1	34 (21.3)
G2	3 (1.9)
*Fibrosis* (*at 6 months*)	
G0	107 (66.9)
G1	15 (9.4)
G2	1 (0.6)
Not available	37 (23.1)
*Breast pain* (*at 6 months*)	
G0	104 (65)
G1	16 (10)
G2	3 (1.9)
Not available	37 (23.1)
*Breast induration tumour bed* (*at 6 m*)	
G0	84 (52.5)
G1	37 (23.1)
G2	2 (1.3)
Not available	37 (23.1)
*Skin hyperpigmentation* (*at 6 months*)	
G0	111 (69.4)
G1	12 (7.5)
Not available	37 (23.1)
*Breast oedema* (*at 6 months*)	
G0	101 (63.1)
G1	21 (13.1)
G2	1 (0.6)
Not available	37 (23.1)

We evaluated late toxicity at 6 months in 123 patients: G1 fibrosis in 15 patients (9.4%) and one patient with G2 (0.6%). Pain was reported as G1 in 16 patients (10%) and G2 in three (1.9%). Grade 1 breast induration in the tumour bed was present in 37 patients, whereas two had G2 (1.3%). There were 12 patients (7.5%) with G1 skin hyperpigmentation, G1 oedema occurred in 21 patients (13.1%) while one patient experienced G2 (0.6%).

Of the 26 patients who received a sequential boost, we have chronic toxicity data for 16 patients. Fourteen patients had no fibrosis and two patients had grade 1 fibrosis. The patient with grade 2 fibrosis did not receive sequential boost. Receiving or not receiving boost was not significantly associated with fibrosis (*p* = 0.9). Induration in the surgical bed was associated with a greater volume of breast PTV (*p* = 0.05).

## 4 DISCUSSION

This descriptive retrospective observational study of ultrahypofractionation in breast cancer shows that use of the FAST‐forward protocol is feasible in early breast cancer within the clinical setting. Our results indicate a very low rate of recurrence, with exclusive local recurrence in 1 patient (0.6%) and a 2‐year OS and DFS of 100% and 98.1%, respectively.

These results are similar in efficacy to recent randomised trials that compared hypofractionated schemes to more extended courses of radiation. The UK FAST trial compared 50 Gy in 2 Gy fractions over 5 weeks with 28.5–30 Gy in 5.7–6 Gy fractions for 5 weeks. At 10 years of follow‐up, the trial found a low recurrence rate in the different groups, with 11 ipsilateral breast cancer events without significant differences between the arms. In terms of normal tissue effects, there was no difference between the 28.5 Gy and 50 Gy regimen, but there was an increase in the effects (shrinkage, induration, telangiectasia, oedema) in the 30 Gy arm (OR 2, 12 [95% CI, 1.55 to 2.89, *p* < 0.001] for 30 Gy and 1.22 [95% CI, 0.87 to 1.72, *p* = 0.248] for 28.5 Gy vs. 50 Gy).^14^ More recently, the “FAST‐Forward” trial compared 40 Gy in 2.67 Gy fractions over 3 weeks with a course of 27 Gy in 5.4 Gy fractions over 1 week or 26 Gy in 5.2 Gy fractions over 1 week. At 5 years, the primary endpoint (ipsilateral breast tumour relapse) was no different among the three arms. Other endpoints such as the incidence of regional relapse, distant relapse, disease‐free survival, and OS were also similar. The 26 Gy scheme proved to be as effective as HF‐RT in 15 sessions for late effects in normal tissue (OR 1.12 [0.94 to 1.34], *p* = 0.20) in contrast to the 27 Gy group, which demonstrated a significantly increased risk of breast‐related effects compared to 40 Gy (OR 1.55 [95% CI 1.32 to 1.83], *p* < 0.0001).^15^ Therefore, the UK FAST trial and the UK FAST‐forward study showed that regimens of 28.5 Gy in 5 fractions for 5 weeks and 26 Gy in 5 fractions over 1 week were not inferior to the conventional treatment of 50 Gy in 25 fractions over 5 weeks and the hypofractionation regimen of 40 Gy in 15 fractions, respectively.

Our results show a very low rate of acute toxicity and an absence of chronic toxicity. These results confirm the low toxicity observed in the UK FAST trial and in the FAST‐Forward trial. Moreover, the low toxicity of our regimen also confirmed the findings of a study that scored skin toxicity with CTCAE criteria among patients treated with 40 Gy in 2.66 Gy fractions over 3 weeks compared with 27 Gy in 5.4 Gy fractions or 26 Gy in 5.2 Gy fractions over 1 week. In the group of patients receiving 26 Gy, no grade 3 toxicity was observed.^20^

Our investigation confirms the results of other retrospective studies conducted with fewer patients. Chaffai et al.^21^ retrospectively analysed 30 patients treated with a fractionated scheme of radiotherapy in five sessions, observing only toxicity due to G1 radiodermatitis and no other relevant toxicity. Similarly, Sigaudi et al.^22^ analysed an ultrahypofractionated regimen in 70 patients, finding G2 radiodermatitis in 6.7%, and G2 induration in 4.4% of the patients, which resembles our data (5% G2 radiodermatitis and 1.3% G2 induration). Ultrafractionated schemes in breast cancer may have important implications in different clinical settings. The COVID pandemic has increased the scientific community's interest in radiotherapy regimens that shorten overall treatment time. Recent international guidelines suggest implementation of the “UK FAST‐forward” scheme adopted during the COVID‐19 pandemic.^23^ In addition, older or frail patients with morbidities, or patients who have to travel a long distance to receive treatment are other clinical factors in which the use of this scheme should be considered. In a recent study, Corrigan et al.^24^ studied the characteristics associated with the use of ultrahypofractionated scheme at their centre. They identified that patients ≥50 years old, low histological grade, exclusion of low axilla from the treatment volume, and longer travel distance were significantly associated with delivering ultrafractionated shorter treatments.

Finally, reducing the fractions in RT treatment for breast cancer may have important economic implications. In a recent study, Yaremko et al.^25^ used cost minimisation analysis to quantify the potential savings associated with hypofractionated regimens. They showed that the FAST‐Forward regimen resulted in the greatest reduction of costs in terms of infrastructure and human resources by 36% and 42%, respectively, compared to the standard.

In recent decades, numerous clinical trials have studied the omission of postoperative radiotherapy treatment in low‐risk breast cancer. The CALGB‐9343^26^ and PRIME II^27^ studies were conducted to determine whether there is a benefit of adjuvant radiotherapy after breast‐conserving surgery and confirmed that the addition of radiotherapy to hormonal therapy improved local control (LC) at 10 years, but without any improvement in OS or DFS. These trials support the omission of radiotherapy in low‐risk breast cancer with hormonal therapy alone with arguments about discomfort, cost and significant short‐ and long‐term toxicity of postoperative treatment with radiotherapy.^28^ However, these trials did not categorise patients based on performance status, did not collect data on coexisting diseases or prospectively monitor adherence to endocrine therapy, and enrolled patients when radiotherapeutic treatment was completely different from the current one, with a total treatment time of 3–6 weeks, with different technology and techniques, and with higher toxicity.^29^ The ongoing clinical trials, such as LUMINA^30^ and IDEA,^31^ are prospective studies aimed at utilising advanced biomarkers to potentially exclude radiotherapy in low‐risk cancer cases. These trials are single‐arm studies without a comparator group: they recommend omission of RT regardless of progression or tolerability and compliance with endocrine therapy. However, omission of RT remains clearly investigable at this stage.^32^ Although all these studies indicated improved local control with radiotherapy in combination with hormonal therapy, the interpretation consistently leaned towards the possibility of omitting radiotherapy. Therefore, we consider that ultra‐hypofractionation in five weekly sessions with new RT techniques in postoperative radiotherapy of breast cancer represents an excellent treatment option for this type of patients, as it has demonstrated high efficacy while significantly reducing treatment‐related burdens, minimising toxicity and even reducing costs compared to endocrine therapy.

In addition, whole breast irradiation after conservative surgery for ductal carcinoma in situ (DCIS) reduces local recurrence.^33^ Randomised trials like DBCG HYPO Trial^7^ confirmed the efficacy and the safety of hypofractionated in 15 fractions. However, the role of ultrahypofraction in patients with DCIS has not yet been clearly. We included an 11.3% of patients with DCIS treated with 26 Gy in five fractions and we did not determine a significant increase in breast induration. These results provide preliminary evidence that postoperative ultrahypofractionated radiotherapy is a viable option for DCIS.

Our study has limitations derived from its retrospective nature. Moreover, the follow‐up is still too short to derive any definitive conclusions about efficacy and long‐term toxicity. However, it has some strength that should be highlighted. Although the number of patients analysed is low, to the best of our knowledge this is the largest retrospective study published in the literature to date. Moreover, all patients have been treated homogeneously by the same physicians, with a very close follow‐up. Finally, all the treated patients in the Department have been consecutively included in the study.

Ultrahypofractionation is a novel regimen of RT which allows treatment to be carried out in five fractions, with important clinical benefits for frail patients or those living far from the hospital. In our experience, treatment with the “UK FAST‐Forward” scheme in low‐risk breast cancer patients with 26 Gy in five fractions within the clinical setting is feasible, reproducing the low rates of recurrence and toxicity observed in the randomised trials.

## AUTHOR CONTRIBUTIONS

**Angel Calvo Tudela:** Data curation (equal); writing – original draft (lead); writing – review and editing (lead). **María Jesús García Anaya:** Data curation (equal); formal analysis (lead); methodology (equal). **Salvador Segado Guillot:** Data curation (equal). **Nuria Martin Romero:** Data curation (equal). **María Jesús Lorca Ocón:** Data curation (equal). **José Antonio Medina Carmona:** Project administration (lead). **Jaime Gómez‐Millán:** Supervision (equal); writing – review and editing (equal). **Isabel García Ríos:** Conceptualization (lead); data curation (equal); investigation (equal).

## Data Availability

Data available on request due to privacy/ethical restrictions The data that support the findings of this study are available on request from the corresponding author. The data are not publicly available due to privacy or ethical restrictions.
